# A Genome-Wide siRNA Screen to Identify Modulators of Insulin Sensitivity and Gluconeogenesis

**DOI:** 10.1371/journal.pone.0036384

**Published:** 2012-05-09

**Authors:** Ruojing Yang, Raul G. Lacson, Gino Castriota, Xiaohua D. Zhang, Yaping Liu, Wenqing Zhao, Monica Einstein, Luiz Miguel Camargo, Sajjad Qureshi, Kenny K. Wong, Bei B. Zhang, Marc Ferrer, Joel P. Berger

**Affiliations:** 1 Department of Metebolic Disorders-Diabetes, Merck Research Laboratories, Rahway, New Jersey, United States of America; 2 Cell Based HTS, Merck Research Laboratories, North Wales, Pennsylvania, United States of America; 3 Biometrics Research, Merck Research Laboratories, West Point, Pennsylvania, United States of America; 4 Department of Guided Solutions, Merck Research Laboratories, Rahway, New Jersey, United States of America; 5 Department of Atherosclerosis, Merck Research Laboratories, Rahway, New Jersey, United States of America; Beckman Research Institute of the City of Hope, United States of America

## Abstract

**Background:**

Hepatic insulin resistance impairs insulin’s ability to suppress hepatic glucose production (HGP) and contributes to the development of type 2 diabetes (T2D). Although the interests to discover novel genes that modulate insulin sensitivity and HGP are high, it remains challenging to have a human cell based system to identify novel genes.

**Methodology/Principal Findings:**

To identify genes that modulate hepatic insulin signaling and HGP, we generated a human cell line stably expressing beta-lactamase under the control of the human glucose-6-phosphatase (G6PC) promoter (AH-G6PC cells). Both beta-lactamase activity and endogenous G6PC mRNA were increased in AH-G6PC cells by a combination of dexamethasone and pCPT-cAMP, and reduced by insulin. A 4-gene High-Throughput-Genomics assay was developed to concomitantly measure G6PC and pyruvate-dehydrogenase-kinase-4 (PDK4) mRNA levels. Using this assay, we screened an siRNA library containing pooled siRNA targeting 6650 druggable genes and identified 614 hits that lowered G6PC expression without increasing PDK4 mRNA levels. Pathway analysis indicated that siRNA-mediated knockdown (KD) of genes known to positively or negatively affect insulin signaling increased or decreased G6PC mRNA expression, respectively, thus validating our screening platform. A subset of 270 primary screen hits was selected and 149 hits were confirmed by target gene KD by pooled siRNA and 7 single siRNA for each gene to reduce G6PC expression in 4-gene HTG assay. Subsequently, pooled siRNA KD of 113 genes decreased PEPCK and/or PGC1alpha mRNA expression thereby demonstrating their role in regulating key gluconeogenic genes in addition to G6PC. Last, KD of 61 of the above 113 genes potentiated insulin-stimulated Akt phosphorylation, suggesting that they suppress gluconeogenic gene by enhancing insulin signaling.

**Conclusions/Significance:**

These results support the proposition that the proteins encoded by the genes identified in our cell-based druggable genome siRNA screen hold the potential to serve as novel pharmacological targets for the treatment of T2D.

## Introduction

Insulin resistance in liver, skeletal muscle, and fat leads to the development of type 2 diabetes (T2D) [1], [2]. In addition, insulin resistance is closely associated with central obesity, dyslipidemia, atherosclerosis, hypertension, and inflammation [3]. Hepatic insulin resistance results in excessive hepatic glucose production (HGP), which plays a major role in the development of hyperglycemia. Conversely, diminution of HGP by various anti-diabetic agents reduces hyperglycemia in humans and preclinical species. The major action of metformin, a first-line T2D therapeutic agent, is to reduce elevated HGP, although the molecular mechanism mediating this beneficial action is not fully understood [4], [5], [6]. Inhibition of glucagon action by glucagon-neutralizing antibodies, antagonistic glucagon peptide analogs, or glucagon receptor (GCGR) anti-sense oligonucleotides inhibit HGP and reduce blood glucose levels in diabetic animals [7], [8], [9], [10], [11]. Additionally, small molecule GCGR antagonists inhibit glucagon-induced increases of blood glucose in humans and animals [12], [13], [14], [15]. Taken together, these results indicate that enhancing hepatic insulin sensitivity and decreasing gluconeogenesis (GNG) suppresses HGP and, therefore, reduces diabetic hyperglycemia.

Insulin suppresses HGP by both direct and indirect means, which then mitigates fasting hyperglycemia, impaired glucose tolerance, and postprandial hyperglycemia [16]. Much has been learned in recent years about the molecular mechanisms modulating the inhibition of HGP by insulin. Liver-specific insulin receptor knockout (LIRKO) mice display complete blockage of the hepatic insulin signaling pathway and fail to suppress HGP in response to treatment with exogenous insulin [17]. LIRKO mice develop severe insulin resistance, hyperglycemia, and hyperinsulinemia. Insulin suppresses the expression of several key GNG regulatory genes, including glucose-6-phosphatase (G6PC), phosphoenolpyruvate carboxylase (PEPCK), and fructose-1,6-bisphosphatase [18], [19]. Several lines of evidence have shown that folk-head transcription factor (Foxo1) binds to the promoter region of several GNG genes to activate their transcription, and this interaction can be blocked by insulin treatment [20], [21], [22]. Insulin triggers the phosphorylation of Foxo1 via the PI3-kinase-dependent Akt pathway resulting in the exclusion of Foxo1 from the nucleus, and consequently, decreased transcription of its GNG target genes [23], [24], [25]. The peroxisome proliferator-activated receptor-γ coactivator-1α (PGC-1α) functions as a master regulator of GNG gene expression in liver [26], binding to and activating Foxo1, hepatocyte nuclear factor (HNF)-4α, and glucocorticoid receptor (GR), and thereby fully activating the transcription of GNG genes [26], [27]. Recent studies have demonstrated that insulin directly inhibits PGC-1α activity through Akt-mediated phosphorylation of the co-activator [28]. Insulin also blocks PGC-1α induction of GNG gene expression by disrupting the interaction of PGC-1α and FoxO1 [27].

To discover novel genes that modulate insulin sensitivity and HGP, we developed a high throughput human hepatoma-based G6PC/PDK4 gene expression assay and used it to screen a library containing synthetic small interference RNA (siRNAs) for 6650 genes encoding druggable protein targets. Additional distinct secondary assays were utilized to confirm our primary hits, and identify those that modulate expression of key GNG genes in addition to G6PC and insulin signaling. Lastly, we demonstrated that the GR antagonist RU-486, which has previously been shown to diminish HGP and hyperglycemia in diabetic animals [29] can suppress G6PC expression in our cell-based assay in a manner comparable to knocking down that receptor.

## Results

**Figure 1:**
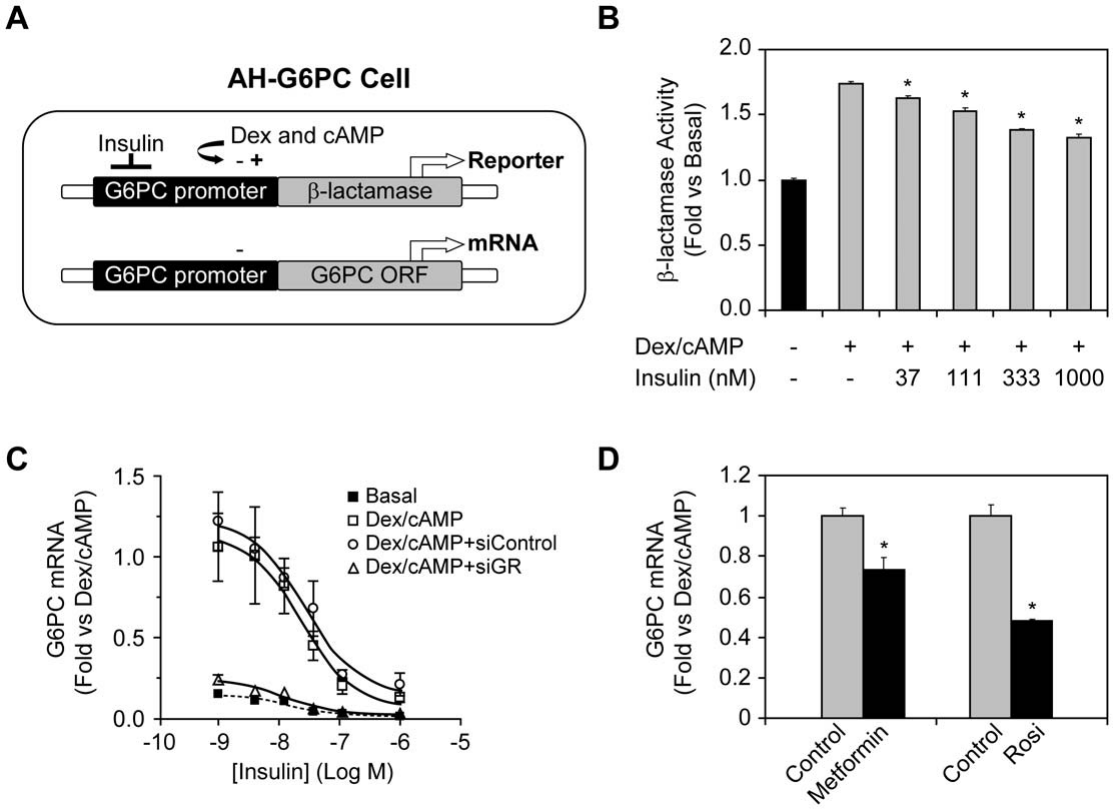
Generation of the hormone-responsive human hepatoma cell line, AH-G6PC, and optimization of assay conditions for siRNA transfection. A. AH-G6PC cells express both the reporter gene, β-lactamase, under the control of the G6PC promoter and endogenous G6PC. B. Insulin dose-responsively decreases Dex/cAMP activation of β-lactamase activity. C. Changes in endogenous G6PC mRNA levels in AH-G6PC cells treated with vehicle (basal), Dex/cAMP, Dex/cAMP after transfection with control siRNAs (siControl), or Dex/cAMP after transfection with glucocorticoid receptor siRNAs (siGR), and increasing concentrations of insulin. D. Incubation of AH-G6PC cells with metformin (667 µM) for 16 h or rosiglitazone (10 uM) for 6 h reduces G6PC mRNA expression. Data are shown as the means ± SEM fold change relative to basal (no Dex/cAMP or insulin) in a study performed in triplicate, and is representative of multiple experiments. *, *P*<0.05 by Student’s t-test vs. Dex/cAMP-treated samples.

To identify novel drug targets that have the potential to enhance insulin sensitivity and decrease HGP, we generated a human hepatoma cell line, AH-G6PC, that stably expressed β-lactamase under the control of the G6PC promoter (Fig. 1A) as described in the “Experimental Procedures” section. This cell line also expressed readily assayable levels of endogenous G6PC mRNA (Fig. 1A). It has previously been demonstrated that G6PC is transcriptionally upregulated by glucagon, catecholamines, glucocorticoids, and downregulated by insulin [30]. Therefore, to examine the hormonal responsiveness of our AH-G6PC cell line, we treated them with 500 nM dexamethasone and a cAMP analogue (pCPT-cAMP) at a concentration of 100 uM (Dex/cAMP) to activate the G6PC promoter and examined the effect of increasing concentrations of insulin on β-lactamase activity and endogenous G6PC mRNA expression. As shown in Fig. 1B, Dex/cAMP increased β-lactamase activity by 1.7-fold compared to basal condition (no Dex/cAMP condition) and insulin dose-dependently suppressed its activity.

While the β-lactamase readout was utilized in developing the basic AH-G6PC cell assay protocol, it did not provide a sufficient dynamic range for use in our high throughput siRNA library screen. In contrast, Taqman analysis showed that Dex/cAMP treatment induced a 10-fold increase in endogenous G6PC mRNA levels, while insulin dose-dependently reduced G6PC mRNA to basal levels with IC_50_ of ∼40 nM (Fig. 1C). Based on these results, we selected Dex/cAMP plus 10 nM insulin as the cell incubation condition for our screen since this concentration of insulin only weakly diminished the robust induction of G6PC expression by Dex/cAMP thereby providing considerable sensitivity to demonstrate potentiation of insulin action by siRNA gene KD. AH-G6PC cells transfected with control siRNA (siControl) responded as expected to Dex/cAMP and insulin in regulating the levels G6PC mRNA, indicating that siRNA transfection *per se* did not negatively affect our assay system (Fig. 1C). GR siRNA (siGR) knocked down GR mRNA by 95% (data not shown) and, as anticipated, led to a large reduction in Dex/cAMP-induced G6PC mRNA expression in cells incubated with vehicle or a wide range of insulin concentrations (Fig. 1C). In additional studies, we found that chronic incubation of AH-G6PC cells with either of the 2 antidiabetic drugs, metformin or rosiglitazone, significantly reduced G6PC mRNA (Fig. 1D) without causing any measurable cytotoxicity (not shown). These data indicated that AH-G6PC cells respond appropriately to metabolic hormones and anti-diabetic drugs known to suppress HGP or enhance insulin sensitivity in T2D patients.

**Figure 2:**
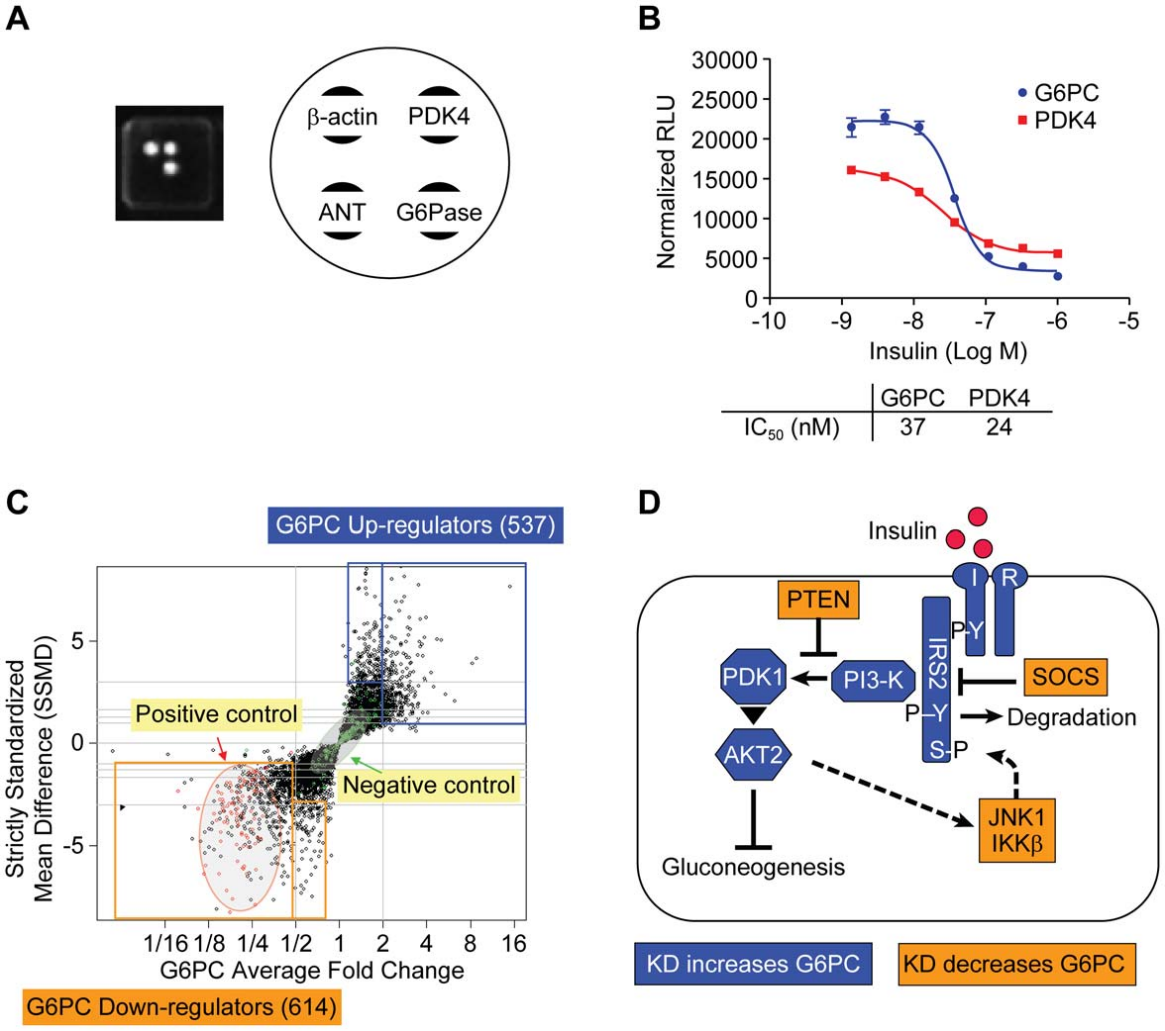
Screening of a druggable siRNA library with a 4-gene High-Throughput-Genomics (HTG) assay. A. Layout of genes within each well of a 4-gene HTG 384-well ArrayPlate. B. Insulin dose-responsively inhibits Dex/cAMP induction of G6PC and PDK4 mRNA expression in AH-G6PC cells when assayed by the HTG platform. Data are presented as the means ± SEM of a study performed in triplicate; similar results were obtained in 4 independent experiments. C. Dual-flashlight plot comparing SSMD values vs. average fold change in G6PC mRNA expression by all siRNA pools in the library in order to select hits that regulate G6PC mRNA levels. Glucocorticoid receptor (GR) siRNAs were used as positive controls in each plates (red dots), while non-targeting siRNAs were used as negative controls in each plate (green dots). The blue square and the small rectangle represent the up-regulators with G6PC ≥2 and SSMD ≥1 and G6PC ≥1.3 and SSMD ≥2, respectively. The orange square and the small rectangle represent the down-regulators with G6PC≤1/2 and SSMD≤−1 and G6PC≤0.7 and SSMD≤−2, respectively. D. Major proximal molecular mediators and key negative modulators of hepatic insulin signaling. siRNA knockdown of target genes that lowered and increased G6PC mRNA expression are indicated in orange and blue, respectively.

To screen our siRNA library, we developed a multiplex High Throughput Genomics (HTG) array that could concomitantly measure the mRNA levels of 4 genes in each well of a 384-well plate. In addition to G6PC, we selected pyruvate dehydrogenase kinase 4 (PDK4) as another test gene, β-actin as the housekeeping gene with which to normalize the above test gene data, and a plant gene, ANT, as the negative control (Fig. 2A). PDK4 functions as a metabolic switch gene that phosphorylates and inhibits pyruvate dehydrogenase which, in turn, increases GNG and decreases glycolysis. PDK4 is upregulated in diabetic or fasting animals, and is downregulated by insulin or refeeding [31]. Dex/cAMP-induced AH-G6PC cell expression of both G6PC and PDK4 mRNA was dose-dependently reduced by insulin in our HTG assay (Fig. 2B) with similar IC_50_s of 24 and 37 nM, respectively. However, the assay window of PDK4 (3-fold) was substantially smaller than that of G6PC (10-fold) due to diminished effects by both Dex/cAMP and insulin on expression of the former gene relative to the latter (Fig. 2B). Thus, we selected G6PC as the primary readout to identify siRNA screen hits, while PDK4 was used to remove preliminary hits that regulated G6PC and PDK4 expression in opposite directions, since they could have unpredictable effects on HGP. β-actin expression was used not only to normalize changes in G6PC and PDK4 expression but also to identify and remove cytotoxic siRNAs since diminished expression of β-actin correlated well with cytotoxicity (not shown). We screened an siRNA library containing pooled siRNAs to each of 6650 druggable genes (3 distinct siRNAs/gene) and defined a primary hit as any gene whose siRNA pool caused a ≥30% increase or reduction in G6PC mRNA expression and an SSMD greater than a certain absolute value (See the Section of Statistical Analysis for more details). Using this approach, our screen identified 614 and 537 hits that decreased or increased G6PC mRNA levels, respectively (Fig. 2C).

Pathway analysis was performed on the above hits as described in the “Experimental Procedures” section and we found that the insulin signaling pathway contained the greatest number of siRNA target genes that regulated G6PC expression. It is well-documented that insulin binds to the hepatocyte insulin receptor (INSR) to increase tyrosine phosphorylation of INSR and insulin receptor substrates (IRS). Tyrosine-phosphorylated IRS proteins bind to and activate phosphatidylinositol 3-kinase (PI3K), which increases phosphatidylinositol-3,4 (PtdIns3,4)P2) and phosphatidylinositol-3,4,5 (PtdIns3,4,5)P3) levels that, in turn, activate phosphoinositide-dependent kinase 1 (PDK1). PDK1 then phosphorylates and activates the kinase Akt, which subsequently suppresses GNG (Fig. 2D). As shown schematically in Figure 2D and quantitatively in Table 1, transfection of siRNAs targeting INSR, IRS2, PDK1, PI3-K subunits (p85β or pl10α), or AKT2 significantly upregulated G6PC mRNA expression (1.6- to 2.2-fold). Phosphatase and tensin homologue (PTEN) is a phosphatase that inhibits the insulin pathway by dephosphorylating PtdIns(3,4,5)P3 to PtdIns(4,5)P2. PTEN siRNAs significantly reduced G6PC mRNA levels in AH-G6PC cells (Table 1). Suppressor of cytokine signaling protein 1 and 3 (SOCS1 and SOCS3) bind to INSR and IRS thereby inhibiting hepatocyte insulin signaling; their siRNAs significantly reduced G6PC expression (Table 1). Insulin signaling activates mammalian target of rapamycin (mTOR) and ribosomal protein S6 kinase (p70S6K), which then activates several downstream serine kinases including c-Jun N-terminal kinase (JNK1), NF-κB inhibitor kinase beta (IKKβ), and protein kinase C (PKC) all of which inhibit insulin signaling by serine phosphorylation of IRS [32]. Our screening results demonstrated that mTOR, JNK1, IKKβ, and atypical protein kinase C-zeta (PKCζ) siRNAs significantly reduced G6PC mRNA levels, consistent with their negative regulatory role on insulin signal transduction. Taken together, these results substantiated our screening platform as a means to identify genes that potentiate insulin sensitivity and inhibit HGP.

**Table 1:** Known mediators and inhibitors of insulin signal transduction identified in the HTG Screen.

	Symbol	Gene_ID	G6PC mRNA (Fold vs. siControl)	PDK4 mRNA (Fold vs. siControl)
**Insulin signaling components**	INSR	3643	1.61*	1.88*
	IRS2	8660	2.08*	1.36*
	AKT2	208	2.03*	0.88
	PDK1	5170	2.09*	1.44*
	p110-alpha	5290	2.09*	1.72*
	p85-beta	5296	2.23*	1.3*
**Negative regulators of insulin signaling**	PTEN	5728	0.36*	0.71*
	SOCS1	8651	0.67*	0.7*
	SOCS3	9021	0.76*	0.52*
	mTOR	2475	0.51*	0.82*
	JNK1	5599	0.55*	0.76*
	IKKbeta	3551	0.61*	1.08
	PKC-zeta	5590	0.6*	0.69*

* Gene expressios are significantly increased or decreased compared to siControl treated samples with p<0.05.

### Hit Confirmation

Our initial screen identified 614 hits whose siRNAs down-regulated G6PC mRNA expression by more than 30%. Utilizing available literature and databases, we determined the known biological functions, human disease associations, and druggability for all of these genes and selected 270 targets for further confirmation based on their superior characteristics.

**Figure 3:**
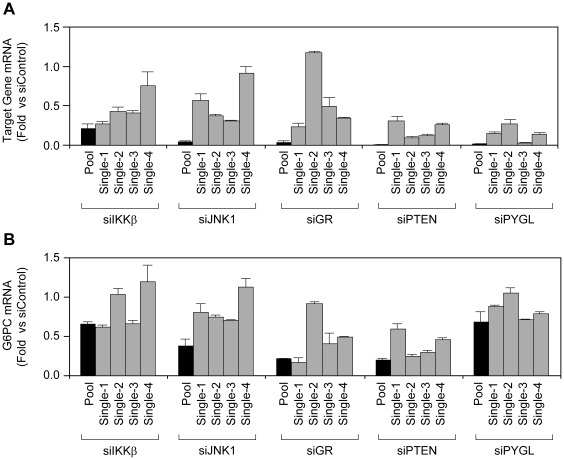
Pooled siRNAs demonstrate greater knockdown efficacy than single siRNAs. Comparison of the ability of transfected pooled vs. single siRNAs to A. knockdown target gene mRNA levels and B. reduce G6PC mRNA levels in AH-G6PC cells incubated with Dex/cAMP and 10 nM insulin for 6 h. Black bars are pooled siRNA samples; grey bars are single siRNA samples. Data are presented as fold changes relative to mRNA expression in siControl-transfected cells and are the means of 3 independent experiments for A and B.

The most commonly used strategy to confirm primary hits from a pooled siRNA screen is to reproduce the results using several distinct single siRNAs. The concept behind this strategy is that if 2 or more different siRNAs for the same gene reduce G6PC mRNA expression, the chance that this is an off-target effect is considerably reduced and, therefore, the hit is confirmed. To evaluate the single siRNA strategy for the next step in the confirmation of our primary hits, we compared the ability of pools containing 4 siRNAs vs. sets of 4 single siRNAs to knock down the expression of several representative target genes and downregulate G6PC mRNA levels. As shown in figure 3A, KD of IKKβ, JNK1, PTEN, GR, and glycogen phosphorylase (PYGL) mRNAs by pooled siRNAs was much more robust than by any single siRNA. With regard to G6PC expression, we found that the pooled siRNAs for IKKβ, JNK1, GR, PTEN, and PYGL were able to reduce G6PC mRNA levels by ≥30% (Fig. 3B). There were at least 2 single siRNAs for IKKβ, GR, and PTEN that reduced G6PC by ≥30%. However, none and only 1 of the single siRNAs for JNK1 and PYGL, respectively, were able to reduce G6PC mRNA by ≥30%. Furthermore, we tested additional 8 hits from the screen for the target gene knockdown and G6PC mRNA reduction in AH-G6PC cells. Consistently, the pooled siRNAs knocked down target genes better than each single siRNA, which, in turn, led to a greater reduction in G6PC mRNA expression by the pooled *vs.* the single siRNAs. Among the 8 genes we tested, there were 2 genes that had 2 single siRNAs reducing G6PC mRNA by ≥30%, 3 genes that had 1 single siRNAs reducing G6PC mRNA by ≥30%, and 3 genes without a single siRNA lowering G6PC mRNA by ≥30%. However, 7 out of 8 genes had more than 2 single siRNAs lowering G6PC mRNA by ≥20%. These data suggest that the reduced knockdown potency by single siRNAs compared to the pooled siRNAs led to less reduction of G6PC mRNA in AH-G6PC cells. Since we were using a 30% reduction in G6PC mRNA as the minimum cutoff for hit selection, the above data `indicated that using single siRNAs would have likely resulted in numerous false negatives (e.g. JNK1 and PYGL) due to ineffective KD of the target genes. Therefore, we used a pooled not a single siRNA protocol in our first hit confirmation assay.

**Figure 4:**
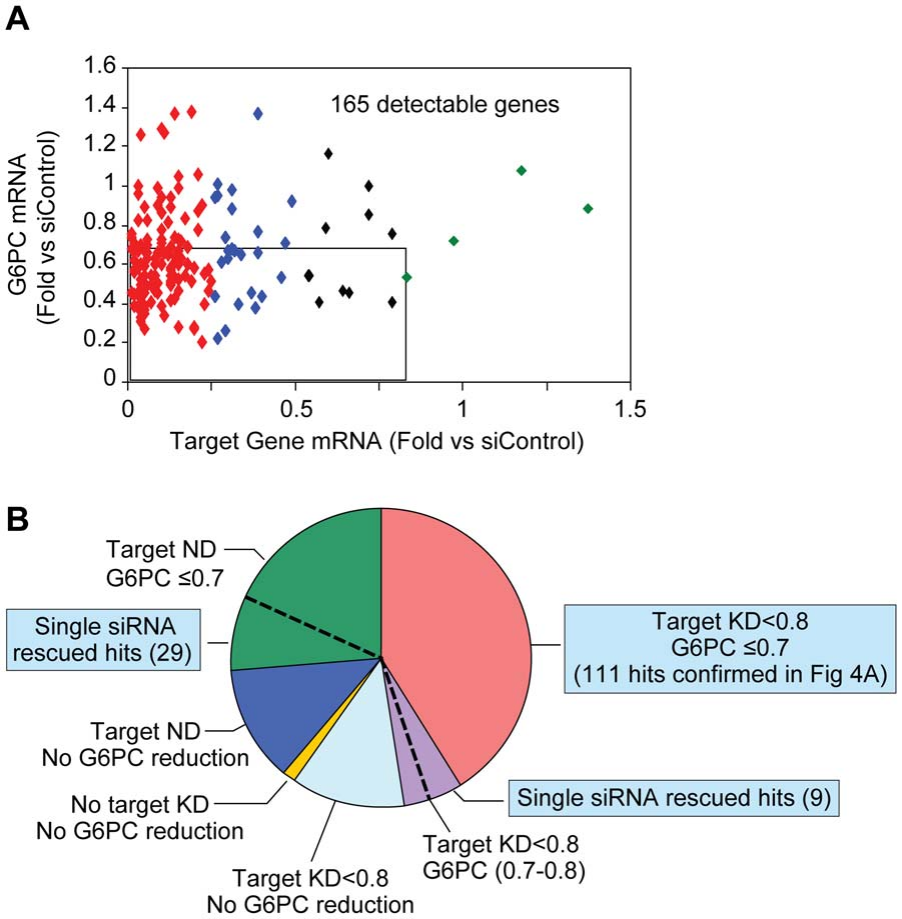
Confirmation of primary screen hits by 2 distinct siRNA assays. A. Pooled siRNA knockdown of 165 Taqman-detectable primary hits vs. reduction of G6PC mRNA expression as determined by Taqman qPCR analysis. Data are shown as fold change compared to mRNA expression in siControl-transfected cells and are the means of a study performed in triplicate. The square contains 111 primary hits that reduce G6PC and target gene expression by ≤0.7- and ≤0.8-fold, respectively. Red, blue, black, and green dots represent target gene knockdown by <0.25-fold, between 0.25-fold to 0.5-fold, between 0.5-fold to 0.8-fold, and no knockdown, respectively. B. Schematic diagram for the 270 primary screen hits separated into different categories after target gene knockdown study. Red represents the 111 hits identified above in A. Blue, orange, and light blue represent primary hits that were not confirmed due to lack of G6PC knockdown. Sets of 7 distinct single siRNAs to each of the 270 primary screen hits were tested for their ability to diminish G6PC expression using the 4-gene HTG platform to quantify mRNA levels. Any target gene for which ≥2 single siRNAs reduced G6PC mRNA was deemed “rescued”. This assay rescued 9 primary hits whose siRNA knocked down their target gene mRNA but only reduced G6PC mRNA between 0.7- to 0.8-fold (purple) and 29 primary hits that were undetectable by Taqman analysis in the pooled siRNA confirmation assay described in A, but were detectable using enriched cDNA as describe in the “Results” section (green). The blue squares label the gene subsets that were combined to become our final 149 confirmed hits using the confirmation strategy described here. ND: undetectable by Taqman analysis; KD: knockdown.

To confirm the primary hits, it was decided that a target gene would have to be observably expressed in AH-G6PC cells and its measurable KD associated with a reduction in G6PC mRNA expression. Therefore, we developed a high-throughput Taqman qPCR analysis protocol to confirm target gene KD as well as diminished G6PC expression using the same mRNA samples. Specifically, we transfected pooled siRNA into AH-G6PC cells cultured in 96- instead of 384-well plates to increase the amount of mRNA available for Taqman assays of the 270 primary hits. Nevertheless, we could only detect the expression of 165 genes, of which 126 (red) and 25 (blue) genes were knocked down by >4-fold and between 2- to 4-fold, respectively, by their siRNA pools. There were only 4 sets (green) of pooled siRNAs that did not KD their target genes and 10 (black) that knocked down their target genes by 1.2- to 2-fold. With regard to the siRNA pools targeting the 165 detectable genes, 112 reduced G6PC expression by ≥30%, among which 106 and 5 knocked down their target gene by >2-fold and 1.2- to 2-fold, respectively (Fig. 4A). These 111 hits were considered confirmed. Additionally, 14 hits whose siRNAs caused ≥2-fold KD of their target gene but only a 20–30% reduction in G6PC mRNA expression were selected as back-up hits.

Since the amount of mRNA that could be extracted from cells plated in 96-well was limited, the inability to detect 105 primary hits above by Taqman analysis was likely due to their relatively low expression levels. Indeed, when we used highly enriched mRNA prepared from AH-G6PC cells cultured in T75-flasks in our qPCR reactions, we confirmed that 254 of the 270 primary hits were expressed. Amongst the pooled siRNAs of the 105 primary hits that were undetectable when analyzing mRNA extracted from cells in 96-well plates, 71 reduced G6PC mRNA by ≥30%; this gene cohort was therefore chosen as back-up hits. In sum, our first confirmation assay verified 111 hits that markedly reduced both target gene and G6PC expression and identified 85 back-up hits that would need to be “rescued” in a subsequent assay to be considered confirmed.

To rescue target genes from amongst the above 85 back-up hits, AH-G6PC cells were transfected with 7 distinct single siRNAs for each of the 270 primary hits and changes in G6PC mRNA expression were assayed with the previously described 4-gene HTG ArrayPlate. We found that at least 2 single siRNAs for 192 of these 270 hits reduced G6PC expression in this assay (data not shown). Based on these results, we were able to rescue 9 hits from amongst the 14 back-up genes that reduced G6PC mRNA by 20–30% in the pooled siRNA confirmation assay and 29 hits from amongst the 71 back-up genes that were undetectable in the pooled siRNA confirmation assay using mRNA extracted from 96-well plate (Fig. 4B). Therefore, in total, we selected 149 primary hits for further investigation including 111 from the pooled siRNA Taqman assay and 38 from the subsequent single siRNA 4-gene HTG assay.

### Identification of GNG Inhibitors and Insulin Sensitizers

**Figure 5:**
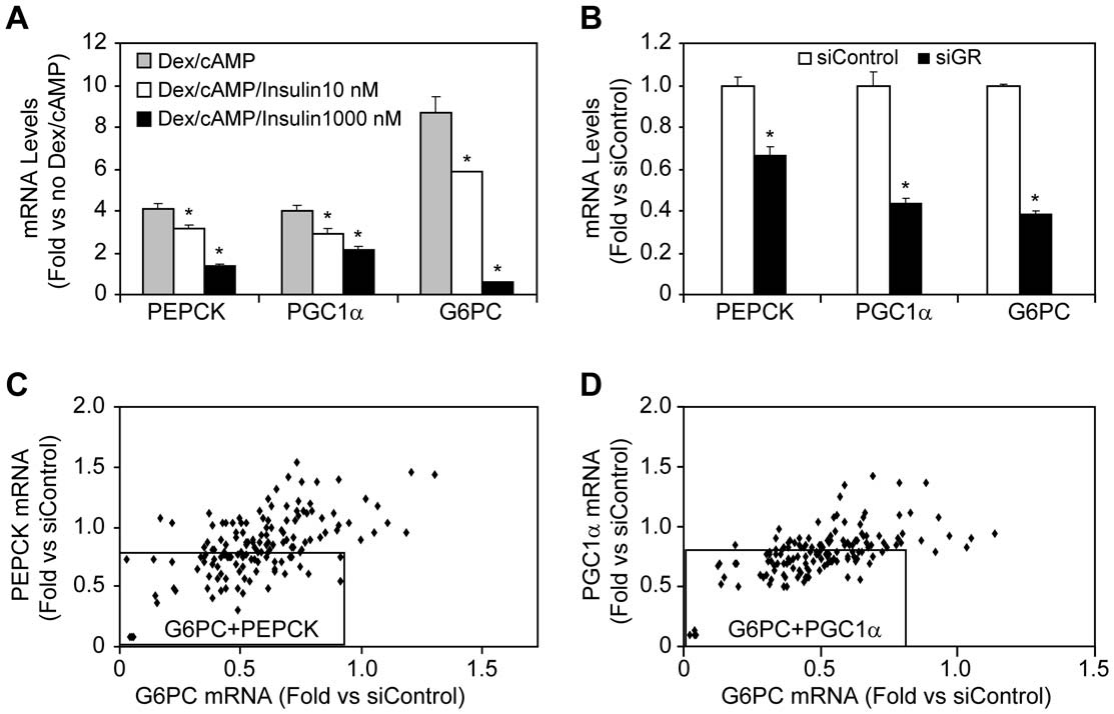
Use of a multiplex HTG assay to identify confirmed hit siRNA pools that decrease expression of key gluconeogenic genes in addition to G6PC. A. Dex/cAMP increases and insulin dose-dependently reduces AH-G6PC cell PEPCK, PGC1α, and G6PC mRNA expression. Data are presented as the means ± SEM of fold change relative to untreated cells in 3 independent experiments. B. Knockdown of GR by siRNA significantly reduces PEPCK, PGC1α, and G6PC mRNA in AH-G6PC cells incubated with Dex/cAMP and 10 nM insulin. Data are presented as the means ± SEM of fold change relative to mRNA expression in siControl-transfected cells in 3 independent experiments. C. Subsets of pooled siRNAs targeting confirmed hits regulate G6PC and PEPCK, or D. G6PC and PGC1α mRNA expression, in AH-G6PC cells treated with Dex/cAMP and 10 nM insulin. Data in Figures 5C and 5D are plotted as fold change relative to mRNA expression in siControl-transfected cells and are the means of 3 independent experiments. The squares contains siRNAs that reduced both G6PC and PEPCK (C) or G6PC and PGC1α (D) expression by 20% and 15%, respectively.

To investigate which of the 149 confirmed hits regulate expression of key GNG genes in addition to G6PC, we developed a 96-well HTG assay for G6PC, PEPCK, and PGC1α, once again β-actin and ANT served as controls. Similar to G6PC, PEPCK and PGC1α expressions in AH-G6PC cells were markedly increased by Dex/cAMP and dose-dependently suppressed by insulin (Fig. 5A). To be consistent with the primary screen, we performed this 5-gene HTG assay using the same Dex/cAMP/insulin treatment protocol and the primary screen siRNAs. The activities of siRNA pools for each of the 149 hits were determined in the assay. KD of the positive control, GR, reduced G6PC, PGC1α, and PEPCK, mRNA expression by 0.62, 0.57 and 0.34-fold compared to negative control, respectively (Fig. 5B). Because the Dex/cAMP-induced assay windows for PEPCK and PGC1α mRNAs were smaller than that for G6PC, we used 15% and 20% reductions in PEPCK or PGC1α and G6PC, respectively, as the cutoffs for hit selection. Of the 149 pooled siRNAs tested, 140 reduced G6PC expression ≥20%; of these, 76 and 99 also reduced PEPCK and PGC1α mRNA levels ≥15%, respectively (Figs. 5C, 5D). Since 62 of the pooled siRNAs inhibited expression of all 3 genes, there were 14 siRNAs that only reduced G6PC and PEPCK and 37 siRNAs that only reduced G6PC and PGC1α. Together, the gene targets of these 113 siRNA pools that reduced the expression of G6PC as well as PEPCK and/or PGC1α were selected as our cohort of negative GNG gene regulators.

**Figure 6:**
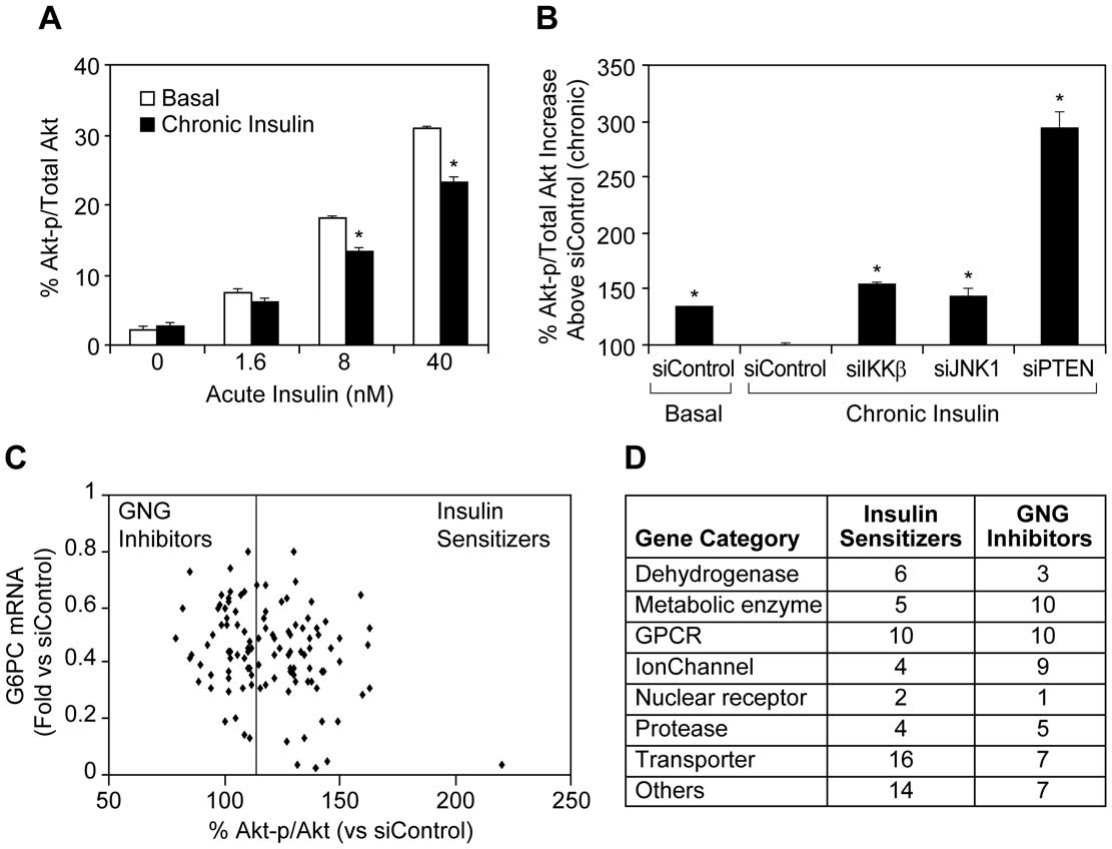
Identification of insulin sensitizer and GNG inhibitor genes. A. Chronic (16 h) 100 nM insulin pretreatment reduces acute (15 min) insulin induction of Akt phosphorylation (Akt-p) in AH-G6PC cells. Akt-p data are presented as the means ± SEM of % Akt-p/total Akt from a representative experiment performed in triplicate. B. Pooled siRNAs targeting JNK1, IKKβ, or PTEN significantly potentiates acute insulin-induced Akt-p after chronic insulin preincubation. Akt-p data are presented as the % Akt-p/total Akt relative to siControl-transfected cells (set as 100%) in response to acute insulin treatment after chronic insulin preincubation and are the means ± SEM of a representative experiment performed in triplicate. C. Differential impact of pooled siRNAs targeting each of the 113 genes described in figures 5C and 5D in regulating G6PC mRNA expression vs. potentiating insulin-induced Akt-p. Hits were separated into insulin sensitizers and GNG inhibitors that augmented acute insulin induction of Akt-p by more and less than 15% (vertical line), respectively. Akt-p data are presented as the % Akt-p/total Akt relative to siControl-transfected cells (set as 100%) in response to acute insulin treatment after chronic insulin preincubation and are the means of 3 independent experiments. G6PC mRNA expression data were obtained from figures 5C and 5D. D. Table summarizing the number of insulin sensitizer and GNG inhibitor hits in distinct druggable gene categories.

To investigate a potential mechanism by which the above 113 hits may have downregulated GNG gene expression, we developed an Akt phosphorylation (Akt-p) assay to identify genes that enhance insulin signaling in AH-G6PC cells when knocked down by transfected siRNA. Chronic hyperinsulinemia has been shown to reduce acute insulin induced Akt-p in hepatocytes [33]. The multiplex electrochemiluminescent ELISA assay described in the “Experimental Procedures” section was used to quantify the percentage of Akt-p vs total Akt (Akt-p%) that served as our readout of insulin signaling. Acute (15 minute) insulin treatment of AH-G6PC cells cultured in basal medium dose-dependently increased Akt-p%, while cells cultured overnight with 100 nM insulin (chronic insulin) became insulin resistant, displaying significantly reduced Akt-p% in response to increasing concentrations of acute insulin (Fig. 6A). Cells pre-incubated in basal media responded to acute 10 nM insulin with a 35% greater increase in Akt-p% than those that had undergone chronic insulin pre-treatment. Cells transfected with siRNA pools targeting the 113 anti-gluconeogenic genes described above were cultured in insulin overnight, washed, and subsequently treated acutely with insulin. As expected, KD of known mediators of insulin resistance, including PTEN, IKKβ, or JNK1, increased Akt-p% by 193%, 54% and 44%, respectively (Fig. 6B). Amongst the pooled siRNAs for the 113 negative GNG gene regulators we assayed, 61 increased Akt-p% by ≥15%, supporting the conclusion that KD of these genes suppressed expression of GNG genes, at least partly, by reversing insulin resistance and potentiating insulin signaling. Therefore, these 61 hits were defined as “insulin sensitizers” while the remaining 52 hits were defined as “GNG inhibitors” (Fig. 6C). Figure 6D quantitatively summarizes the wide variety of druggable gene categories found within the insulin sensitizer and GNG inhibitor target groups identified by our siRNA screening procedure.

**Figure 7:**
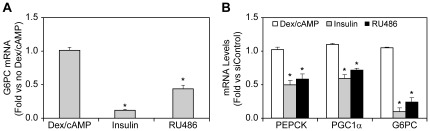
Pharmacological inhibitor of glucocorticoid receptor (GR) led to similar reduction of gluconeogenesis gene expression to GR siRNA in AH-G6PC cell. AH-G6PC cells were preincubated with or without the GR antagonist RU486 (5 µM) for 18 h. The cells were then incubated with Dex/cAMP alone, Dex/cAMP plus 400 nM insulin, or Dex/cAMP plus RU486 (in the cells treated with RU486 overnight) for 6 h. A. Taqman analysis was performed on mRNA isolated from the cells to assay G6PC mRNA relative to Dex/cAMP treated cells. B. Multiplex HTG ArrayPlate analysis of mRNA isolated from the cells was used to quantify PEPCK, PGC1α, and G6PC mRNA expression. Data are plotted as fold change vs. mRNA levels in cells treated only with Dex/cAMP and are the means ± SEM of 3 independent experiments.

The siRNA screen described above identified 113 genes that regulate the expression of key GNG genes. Therefore appropriate pharmacological regulation of the proteins encoded by these genes may reduce HGP and hyperglycemia in T2D patients. To further evaluate the ability of our screen to identify targets that can be pharmacologically manipulated to obtain anti-diabetic activity, AH-G6PC cells were incubated with Dex/cAMP as in our siRNA screen and co-treated with saturating concentrations of insulin (400 nM) or the GR antagonist RU-483 (5 µM). Using the same treatment conditions as the primary siRNA screen, Taqman analysis of G6PC mRNA showed that RU486 significantly lowered G6PC mRNA by 55%, while insulin reduced G6PC mRNA by 90% (Fig. 7A). RU486 at 5 uM also reduced β-lactamase activity in the cells, confirming a reduction in G6PC promoter activity (data not shown). Gene expression analysis using our 5-gene HTG assay platform showed that RU486 robustly reduced G6PC, PEPCK and PGC1α expression in a manner similar to insulin (Fig. 7B) and siRNA KD of GR as shown previously in figure 5B. These results are in line with those demonstrating that RU486 lowers hyperglycemia in diabetic mice [29]. These data support the conclusion that pharmacological effectors of the insulin sensitizer and anti-gluconeogenic targets unveiled by the siRNA screening process reported here hold the potential to serve as novel antidiabetic therapeutic agents.

## Discussion

**Figure 8:**
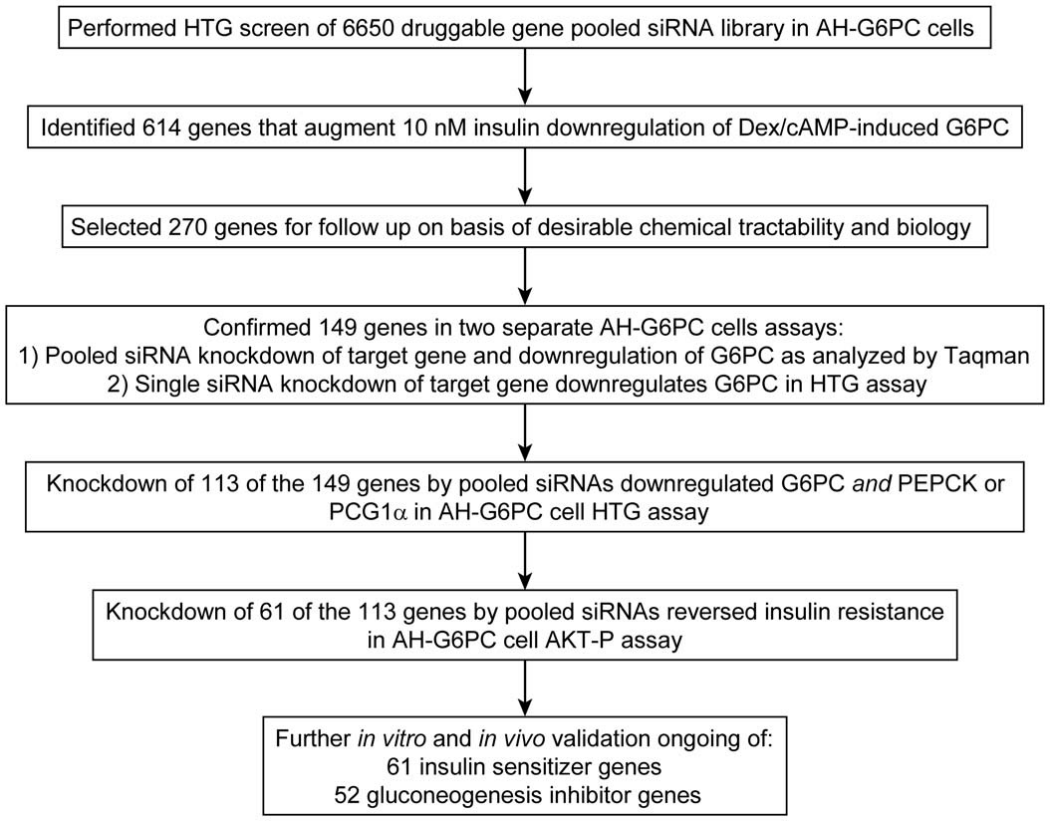
Schematic summary of the siRNA screening process that identified, confirmed, and characterized novel insulin sensitizer and GNG inhibitor target genes.

In order to aid in the discovery of new pharmacological agents that promote insulin action and inhibit HGP, it is necessary to continue identifying genes encoding druggable proteins that regulate insulin sensitivity and GNG. To find such genes, we developed a novel hormonally-responsive human hepatoma cell line, AH-G6PC, and used it, together with a 4-gene HTG ArrayPlate mRNA assay, to screen the pooled siRNAs comprising a 6650 druggable gene library for their impact on the expression of 2 key GNG regulatory genes, G6PC and PDK4. Our initial screen identified 614 primary hits that reduced G6PC mRNA expression without increasing that of PDK4 in AH-G6PC cells treated chronically with Dex/cAMP and insulin (Fig. 8). On basis of their desirable chemical tractability and biology, we selected 270 of these genes for further confirmation in 2 additional siRNA assays using AH-G6PC cells treated pharmacologically as in our primary screen. First, we developed a high-throughput Taqman qPCR assay to determine the impact of the 270 pooled siRNAs on target gene KD and G6PC mRNA expression in the same cellular mRNA samples and used it to confirm 111 primary hits. We also identified a cohort of 85 backup hits whose siRNA pools had a notable but diminished inhibitory effect on G6PC mRNA expression or that were expressed at relatively low levels in comparison with our primary hit genes. Second, using our 4-gene HTG ArrayPlate assay, we examined the effect of single siRNA sets for each of our 270 primary hits on G6PC mRNA expression and thereby rescued 38 of the backup hits described above. Thus, together, these two assays confirmed 149 primary hits for further investigation. To explore the ability of these targets genes to regulate expression of key GNG genes in addition to G6PC, we developed a second multiplex HTG ArrayPlate that allowed us to analyze G6PC, PEPCK, and PGC1α expression in mRNA obtained from AH-G6PC cells treated with pooled siRNAs as well as Dex/cAMP and insulin as in our primary screen. We found that amongst the siRNAs for the 149 confirmed hits, 113 downregulated G6PC and at least 1 of the 2 other GNG genes. We then assayed the ability of pooled siRNAs targeting these 113 genes to augment insulin signaling in AH-G6PC cells that were incubated chronically with insulin to induce insulin resistance as determined by a diminution in acute insulin induction of Akt-p%. We discovered that 61 siRNA pools potentiated acute insulin-stimulated Akt-p%, thereby suggesting that their downregulation of GNG genes was at least partially mediated by their enhancement of insulin signaling. Finally, we demonstrated that 2 *bona fide* pharmacological inhibitors of GNG, the GR selective antagonist RU486 and insulin, decreased Dex/cAMP induction of G6PC, similarly to knocking down expression of GR or genes that negatively regulate insulin signaling, thereby verifying the ability of our screening procedure to identify druggable molecular targets and their pharmacological effectors that can diminish GNG gene expression and augment insulin action.

The screening of genome-wide siRNA libraries has become a powerful tool to identify novel targets for the treatment of different diseases [34]. Because most such screens generate hundreds of primary hits, it is critical to discern the true hits and reduce the number of false positives. The most commonly used method to verify primary hits identified in screens utilizing siRNA pools, is to remove “off-target” hits by testing the activity of sets of multiple single siRNAs targeting each of these genes. Hits are then generally considered confirmed if 2 or more single siRNA reproduce the assay readout obtained in the primary screen. In this way, primary hit genes selected due to off-target effects by a single member of the pooled siRNA sets should be largely eliminated. However, when we performed a pilot study using groups of 4 single siRNAs targeting a number of primary hits that are proven effectors of insulin signaling and/or GNG, we found that this approach did not confirm all of these genes as hits, i.e., it generated false negatives, due to diminished target gene KD in comparison with pooled siRNAs. Therefore, for our first confirmatory assay, we incubated cells with pooled siRNA sets and then used high throughput Taqman analysis to quantify target gene KD and reduction of G6PC mRNA expression. While many primary hits were verified using this procedure, we also observed that the mRNA of numerous targets could not be detected by Taqman analysis apparently due to their relatively low expression levels and the small amount of mRNA that could be extracted from cells cultured in 96-well plates. However, by using enriched mRNA extracted from AH-G6PC cells cultured in T75 flasks, we were able to increase the sensitivity of our Taqman assay such that we could identify a group of backup hits whose pooled siRNAs had diminished G6PC mRNA expression in our first confirmatory assay and now had detectable gene expression in AH-G6PC cells. To further verify a subset of these backup hits as well as another cohort identified in the first confirmatory assay, we utilized a distinct combination of methods that included sets of 7 single siRNA to KD target gene expression in AH-G6PC cells and 4-gene HTG ArrayPlates to determine changes in G6PC mRNA expression. By only rescuing hits when at least 2 of their single siRNAs decreased G6PC mRNA levels, we felt confident that we were not selecting false positives driven by off-target events. Also, by using single siRNA KD at this step in the hit selection process, we were not so concerned that important target genes would be incorrectly filtered out as we would have been if it had been used earlier when our overall confirmatory data was more limited.

Up until this point, our screening process had identified numerous genes whose siRNAs reduced G6PC mRNA levels in a hormone-responsive hepatoma cell line, thereby suggesting that they might suppress HGP. However, since G6PC is only one of several key genes involved in the regulation of HGP, we decided to further filter our hit list by identifying those whose siRNAs also diminished the expression of 2 other key GNG genes. To accomplish this, we developed a multiplex HTG ArrayPlate assay to quantify G6PC, PEPCK, and PGC1α mRNA expression. Our data showed that ∼75% of the pooled siRNAs tested reduced not only G6PC mRNA expression in AH-G6PC cells, but that of at least one of the other two GNG genes as well. Therefore, we concluded that the genes targeted by this subset of siRNAs were potentially important regulators of GNG and, thus, HGP.

While elevated HGP is one important source of the hyperglycemia observed in T2D patients, another major cause is diminished glucose uptake by skeletal muscle. Both of these diabetic pathobiologies have been shown to result, in large part, from obesity-induced insulin resistance in these tissues. It is therefore reasonable to suggest that anti-diabetic agents with optimal glucose lowering activity would not only suppress HGP by directly inhibiting GNG like metformin and glucagon receptor antagonists, but, preferably, enhance insulin sensitivity in liver and skeletal muscle to both reduce HGP and potentiate insulin-stimulated glucose uptake in the periphery. This hypothesis led us to identify the confirmed hits whose siRNAs not only decreased expression of key GNG genes but also enhanced insulin signaling. Our results indicated that 54% of the pooled siRNA sets targeting our aforementioned anti-GNG hits also potentiated acute insulin stimulation of Akt phosphorylation in AH-G6PC cells made insulin resistant by prior chronic insulin treatment. These 61 unique hits will be given high priority in future efforts to validate novel anti-diabetic targets discovered by our screening procedure.

To further demonstrate the ability of our cell-based siRNA screen to identify novel anti-diabetic drug targets, we examined the impact of the GR antagonist RU486, a compound with proven anti-gluconeogenic and glucose lowering activity in diabetic animals [29], on GNG gene expression. We found that in AH-G6PC cells treated chronically with Dex/cAMP, co-treatment with RU486 reduced expression of G6PC, PEPCK and PGC1α mRNA to levels comparable with those obtained with a saturating concentration of insulin. These pharmacological results are in line with antisense oligonucleotide studies demonstrating that liver-specific KD of GR diminishes hepatic expression of G6PC and PEPCK and lessens hyperglycemia in obese diabetic ob/ob and db/db mice [35]. Antisense oligonucleotides targeted against glucocorticoid receptor reduce hepatic glucose production and ameliorate hyperglycemia in diabetic mice. In additional preliminary studies, we identified small molecule effectors of protein targets encoded by other confirmed target genes that reduced expression of the 3 GNG genes in AH-G6PC cells to a similar extent as RU486 (not shown). Furthermore, in an in vivo pilot experiment one of these compounds, an enzyme inhibitor that was previously unknown to regulate GNG gene expression or glucose homeostasis, was able to significantly mitigate hyperglycemia in db/db mice (not shown). On the basis of these results, we expect that more of the hits identified in our siRNA screen will be validated as novel anti-diabetic targets in diabetic animals using both pharmacological and genetic protocols.

In summary, we have developed and performed a unique druggable genome-wide siRNA screen in a hormonally-responsive human hepatoma cell line that has identified genes whose protein products possess noteworthy potential to serve as novel anti-diabetic drug targets. Ongoing experimentation is currently ongoing to further validate these prospective targets and identify small molecule effectors possessing the appropriate characteristics to be developed as efficacious and safe T2D therapeutics.

## Materials and Methods

### Generation of the AH-G6PC Cell Line

A clone of the Alexander hepatoma cell line (PLC/PRF/5 obtained from ATCC; #CRL-8024) that stably expresses β-lactamase under the control of the human glucose-6-phosphatase (G6PC) promoter was generated as follows. The 2.1 kB human G6PC promoter was PCR amplified (Advantage 2 PCR kit, Clontech) from human genomic DNA using the following primers: 5′-GGC TCG AGA AGA CCA GCC TGG GCA AC-3′ and 5′-CGC TCG AGT GAG TCT GTG CCT TGC C-3′. The reporter plasmid p7XUAS-BlaM-pcDNA3.1 (Aurora Biosciences) was digested with Xho I to remove the 7XUAS sequence and ligated with the PCR amplified human G6PC promoter fragment to generate the reporter plasmid pG6PC-BlaM-pcDNA3.1. Using Lipofectamine 2000 (Invitrogen), pG6PC-BlaM-pcDNA3.1 was transfected into Alexander cells plated into 10 cm^2^ dishes at a cell density of 3.4×10^6^ in Optimem 1 media (Invitrogen). Twenty four hours later the cells were subcultured 1∶4 in MEM Alpha Medium (Gibco) supplemented with 10% fetal bovine serum, 100 units/ml penicillin, 100 ug/ml streptomycin in the presence of 600 ug/ml Zeocin to select for stable transfectants. The LiveBLAzer™ FRET – B/G Loading Kit (Invitrogen) was used to asaay +/−1 uM Dex/100 uM 8-CPT cAMP using CCF2-AM to label the live cells with β-lactamase negative cells with a green fluorescence signal and β-lactamase positive cells with a blue fluorescence signal. FACS sorting analysis was used to identify stable transfectants with blue fluorescence signals. Positive cells were grown up and then subcloned by limiting dilution to generate the cloned AH-G6PC cell line. These cells were maintained in the growth medium (MEM Alpha Medium (Gibco) supplemented with 10% fetal bovine serum, 100 units/ml penicillin, 100 ug/ml streptomycin and 0.15 mg/ml Zeocin), at 37°C in 5% CO_2_.

### Gene Expression, siRNA Transfection, β-lactamase, and AKT Phosphorylation Assays

Individual siGENOME and SMARTpool siRNAs from Dharmacon were used to KD all genes of interest. In 96-well plates, 20,000 AH-G6PC cells/well were reverse-transfected using Dharmafect1 transfection reagent (Dharmacon) following the manual instruction. Briefly, DharmaFect1 (0.25 ul per well) was mixed with 24.75 ul OPTIMEM medium for 5 minutes and then combined with 25 ul OPTIMEM medium with siRNA (0.375 ul of 20 uM stock). The mixture was incubated for 20 minutes and added to each well, and then 100 ul of cell suspension (2×10^5^ cells/ml) in growth medium was added to each well. The final siRNA concentration in the transfection mix was 50 nM. After a 24 h incubation with siRNA, the transfection mix was removed, and the cells were allowed to recover in the growth medium for 5 h. The cells were then serum starved for 16 h, and subsequently treated with 0.5 µM dexamethasone, 100 µM 8-CPT cAMP, and 10 nM insulin in serum-free media for 6 h. Total mRNA or total RNA was isolated from cells using Turbocapture96 mRNA Plates or RNeasy 96-well mini-kit according to protocols provided by the manufacturer, respectively (Qiagen). cDNA was synthesized with the iScript cDNA Synthesis Kit (BioRad). Amplification of each target cDNA was performed with the Taqman PCR Reagent Kit in an ABI Prism 7900 Sequence Detection System (PE Applied Biosystems) with a program of 40 cycles, each cycle consisting of 95°C for 15 seconds and 60°C for one minute. Taqman qPCR data were analyzed using RQ Manager 1.2 (Applied Biosystems), with automatic baseline and manual threshold setting at 0.2 to generate Ct values. Genes with a Ct ≥36 were considered undetectable. The ??Ct method was used to calculate the relative expression level and gene KD. G6PC mRNA levels were normalized by the amount of β-actin mRNA detected in each sample. β-lactamase was measured using the LyticBLAzer_h-BODIPY FL Kit according to protocols provided by the manufacturer (Invitrogen). Akt proteins were analyzed using a multiplex electroluminescence assay kit detecting Akt phosphorylated at Ser-473 and total Akt following the manual instruction (Meso Scale Discovery).

### Primary siRNA Screen and Multiplex Gene Expression using a High-Throughput Genomics (HTG) ArrayPlate

In a 384-well plate, 5000 AH-G6PC cells were reverse-transfected with 20 nM siControl non-targeting siRNA (Dharmacon) and 6650 druggable siRNA pools (3 siRNA/pool) (Sigma Mission human druggable genome) using Dharmafect-1 (Dharmacon) as described previously with 0.1 ul DharmaFect1, siRNAs, and 10 ul OPTIMEM per well. The cells were then incubated at 37°C for 24 h post-transfection, after which the growth media was replaced with MEM Alpha supplemented with 10% serum for 5 h. The cells were subsequently incubated with serum free MEM Alpha overnight. Lastly, the medium was changed to serum free MEM Alpha with 100 µM 8CPT-cAMP, 0.5 µM dexamethasone, and 10 nM insulin (Sigma) for 6 h.

A high throughput multiplex quantitative nuclease protection assay (qNPA) was performed on the treated cells as previously described [36] to simultaneously measure mRNA expression of G6PC, PDK4, β-actin, and a negative control plant gene, ANT. Media was removed from the cell culture plates and a proprietary lysis reagent (High Throughput Genomics) is added (12.5 ul) to lyse the cells, overlayed with denaturation oil (35 ul), A cocktail of nuclease protection probes (NPP, synthetic biotinylated DNA that complements the RNA of the target gene being measured) were added and incubated at 95°C for 10 minutes in oven then cooled down to room temperature and incubated at 50°C for 6 hours. S1 nuclease (10 ul) was added and incubated for 50 minutes at 60°C, during which time all the nonspecific RNA, DNA and excess single stranded nuclease protection probes are destroyed such that only the specific probe/target hybrid duplexes remained. Sodium hydroxide was then added and the sample heated at 95°C for 10 minutes to dissociate the probes from the target RNA and destroy the released target RNA. The solution was neutralized and transferred onto the programmed 96-well 4-gene HTG ArrayPlate (High Throughput Genomics). NPP probes were captured during an overnight incubation at 50°C. The media was removed and a cocktail of NPP-specific detection linkers were added, which hybridized to their respective captured NPP during a 1 hour incubation at 60°C. The array was then washed and a HRP-labeled oligonucleotide detection probe was added and incubated at 37°C for 1 hour; the array was then washed again to remove unbound detection probe. Luminescent peroxidase substrate (Atto-PS™, Lumigen) was added and the plate imaged for 15 sec. to measure the intensity of every element within the plate. Imaging was carried out using the Omix Imager (HTG Molecular), which images all wells of an entire plate at the same time. The intensity from each individual element of the array was measured. The position within the array identified the gene being measured, and the amount of luminescence from each element indicated the amount of each gene. The Omix software “extracts” the data from the images, quantifying each element, and then permitting analysis such as subtraction of background and normalization to β-actin. A second multiplex HTG ArrayPlate assay was used to identify target genes that when knocked down affect expression of PGC1α and PEPCK in addition to G6PC; β-actin, and negative ANT once again served as controls genes. The assay was performed in the same manner as described above.

### High Throughput Taqman Analysis of siRNA-mediated Gene Knockdown

AH-G6PC cells were transfected with pooled siRNA (3/gene) to each of 270 primary assay gene hits in 96-well plates. After transfection and treatment of the cells as described above, mRNA was extracted followed by cDNA synthesis to prepare the samples for Taqman qPCR analysis. For test gene KD analysis, cells transfected by a negative control siRNA (siControl) were processed in parallel and used as a calibrator for calculating the level of test gene KD. Two hundred seventy distinct Taqman gene expression assays and Gene Expression Master Mix (Applied Biosystems) were custom pre-plated in 384-well PCR plates, with the Taqman assay well locations matching the locations of corresponding siRNA samples. β-actin Taqman assay (Applied Biosystems) was also pre-plated in 384-well PCR plates for measuring β-actin cDNA in all samples. For each primary hit, we ran Taqman qPCR of the target gene and β-actin control in triplicate for both siRNA- and siControl-transfected samples. For Taqman qPCR reactions, 2 ul of cDNA was transferred to the 8 ul Taqman assay and Gene Expression Master Mix using Biomek FX liquid handler (Beckman Coulter). PCR amplifications and fluorescence detection was conducted in an Applied Biosystems 7900HT sequence detector with a program of 40 cycles as described above.

### Statistical Analysis of Primary siRNA Screening Data

Data quality was assured through implementing quality control procedures as described previously [37]. We calculated the average fold change and strictly standardized mean difference (SSMD) for all siRNAs (Zhang, 2011). As previously described, SSMD is the ratio of mean and standard deviation of a difference [38]. We then selected hits using a dual-flashlight plot in which both average fold-change and SSMD were considered simultaneously [39]. In the primary screen described previously, we first used the criterion of average fold change in G6PC expression of ≥2 and SSMD ≥1 for selecting up-regulators and average fold change in G6PC mRNA expression of ≤0.5 and SSMD≤−1 for selecting down-regulators. To rescue hits with consistent but not very strong average activity, we further used the criterion of SSMD ≥2 and an average increase of between 30% and 200% in G6PC mRNA expression for selecting additional up-regulators and SSMD≤−2 and an average reduction of between 30% and 200% in G6PC mRNA expression for selecting additional down-regulators (Fig. 2C).
